# p47phox-Dependent Reactive Oxygen Species Stimulate Nuclear Translocation of the FoxO1 Transcription Factor During Metabolic Inhibition in Cardiomyoblasts

**DOI:** 10.1007/s12013-018-0847-4

**Published:** 2018-06-29

**Authors:** Ellis N. ter Horst, Nynke E. Hahn, Dirk Geerts, René J. P. Musters, Walter J. Paulus, Albert C. van Rossum, Christof Meischl, Jan J. Piek, Hans W. M. Niessen, Paul A. J. Krijnen

**Affiliations:** 1 Department of Cardiology, Amsterdam UMC, location AMC, Amsterdam, The Netherlands; 2 Netherlands Heart Institute, Utrecht, The Netherlands; 3 Amsterdam Cardiovascular Sciences, Amsterdam UMC, location VUmc, Amsterdam, The Netherlands; 4 Department of Pathology, Amsterdam UMC, location VUmc, Amsterdam, The Netherlands; 5 Department of Medical Biology L2-109, Amsterdam UMC, location AMC, Amsterdam, The Netherlands; 6 Department of Physiology, Amsterdam UMC, location VUmc, Amsterdam, The Netherlands; 7 Department of Cardiac Surgery, Amsterdam UMC, location VUmc, Amsterdam, The Netherlands; 8 Department of Cardiology, Amsterdam UMC, location VUmc, Amsterdam, The Netherlands

**Keywords:** FOXO1, p47^phox^, Reactive oxygen species, Ischemia, NADPH oxidase

## Abstract

Reactive oxygen species (ROS) control forkhead box O (FOXO) transcription factor activity by influencing their nuclear translocation. However, knowledge of the ROS cellular source(s) involved herein remains scarce. Recently, we have shown p47^phox^-dependent activation of ROS-producing NADPH oxidase (NOX) at the nuclear pore in H9c2 rat cardiomyoblasts in response to ischemia. This localizes NOX perfectly to affect protein nuclear translocation, including that of transcription factors. In the current study, involvement of p47^phox^-dependent production of ROS in the nuclear translocation of FOXO1 was analyzed in H9c2 cells following 4 h of metabolic inhibition (MI), which mimics the effects of ischemia. Nuclear translocation of FOXO1 was determined by quantitative digital-imaging fluorescence and western blot analysis. Subsequently, the effect of inhibiting p47^phox^-dependent ROS production by short hairpin RNA (shRNA) transfection on FOXO1 translocation was analyzed by digital-imaging microscopy. MI induced a significant translocation of FOXO1 into the nucleus. Transfection with p47^phox^-shRNA successfully knocked-down p47^phox^ expression, reduced nuclear nitrotyrosine production, an indirect marker for ROS production, and inhibited the nuclear translocation of FOXO1 following MI. With these results, we show for the first time that nuclear import of FOXO1 induced by MI in H9c2 depends critically on p47^phox^-mediated ROS production.

## Introduction

Mammalian cells produce reactive oxygen species (ROS) in response to a multitude of (patho)physiological conditions and thereby regulate cell fate decisions [1]. In the last two decades it has become recognized that these ROS, rather than being by-products of signaling events, act as second messengers in cellular signaling through the reversible oxidation of cysteine residues in signal transduction proteins, which alters their activity [2, 3]. These signal transduction proteins include protein kinases, phosphatases, and transcription factors. Through redox modification of these proteins, ROS are involved in gene expression regulation and thereby influence processes like cell proliferation, migration, and apoptosis [1, 4].

A number of redox-sensitive transcription factors have been described that are either activated or inactivated through redox modifications, including NF-κB, c-Jun and Nrf2 [4–8]. Another group of transcription factors that has been shown to be under redox control is the forkhead box O (FOXO) family of transcription factors [9], of which four are expressed in humans (FOXO1, FOXO3a, FOXO4, and FOXO6) [10]. FOXO transcription factors are involved cellular functions like cell-cycle arrest, apoptosis, ROS scavenging and DNA repair and influence cell fate decisions [10–14].

FOXO activity regulation is complex and includes phosphorylation/dephosphorylation and redox signaling [9, 15]. Also, FOXO activity critically depends on its subcellular localization. In response to insulin and growth factor signaling, functional nuclear FOXO is deactivated through phosphorylation by the PI3K/Akt pathway, leading to release from its DNA binding sites and export from the nucleus into the cytoplasm [15]. In contrast, oxidative stress can directly oppose the PI3K/Akt pathway and promote FOXO nuclear translocation [16].

ROS are involved in FOXO activity regulation. It was shown in cardiomyocytes that hypoxia/reoxygenation and oxidative stress-induced nuclear translocation of FOXO1, where it promoted cell survival [17]. In C2C12 murine myocytes, ROS scavenger NAC counteracted IGF-I-induced FOXO1 phosphorylation [18]. However, knowledge of the cellular source (s) of these ROS remains scarce.

As ROS-producing enzymes, the family of multi-component NADPH oxidases (NOX) appears to be important in redox signaling [19] and may be linked to FOXO. This has been suggested in human pulmonary artery smooth muscle cells (SMC), where FOXO3a activity was increased by NOX4 over-expression and decreased by NOX4 knockdown [20]. Moreover, Cui et al. [21] showed a significant reduction in Akt and FOXO4 phosphorylation in hepatic stellate cells isolated from NOX1 null-mutant mice compared to cells isolated from wild-type mice [21]. These data indicate a link between NOXes and FOXO. Additionally, we have shown previously in H9c2 rat cardiomyoblasts that the NOX subunits NOX2, p22^phox^ and p47^phox^ are targeted to the nuclear pore in response to ischemia and hyper-homocysteinemia. In the nuclear pore, ROS is produced locally and depends critically on the presence of p47^phox^ [22, 23]. Although it is not fully understood how NOX-derived ROS achieve signal specificity, the subcellular localization and activation of NOX in specific subcellular compartments may be a means to achieve signal specificity. Thus, the activation at the nuclear pore perfectly places NOX to regulate protein translocation in or out of the nucleus, including that of transcription factors. Therefore, we hypothesized that in response to ischemia, p47^phox^ could be critically involved in the nuclear translocation and activation of FOXO proteins in cardiomyocytes. To assess this, we currently analyzed the involvement of p47^phox^-dependent production of ROS in the subcellular translocation of FOXO1 in H9c2 cells in response to metabolic inhibition (MI), which mimics ischemia.

## Materials and Methods

### Cell culture

Rat cardiomyoblasts [24] (H9c2 cells; ATCC, Manassas, VA, USA) were cultured in Dulbecco’s modified eagles medium (DMEM, Cambrex Corporation, East Rutherford, NJ, USA) containing 10% (v/v) heat inactivated fetal calf serum (FCS, BioWhittaker, Walkersville, MD, USA), 100 IU/ml penicillin (Yamanouchi Europe, Meppel, The Netherlands), 100 μg/ml streptomycin (Radiopharma Fisiopharme, Palomonte, Italy) and 2 mM l-glutamine (Invitrogen, Carlsbad, CA, USA). H9c2 cells were cultured at 37 °C in a humidified atmosphere containing 5% CO_2_. To mimic ischemia, H9c2 cells were incubated for 4 h in a MI buffer (0.9 mM CaCl_2_·H_2_O, 106 mM NaCl, 3.8 mM NaHCO_3_, 4.4 mM KCL, 1 mM MgCl_2_·H_2_O, pH 6.6), containing 20 mM (2-deoxy)glucose to impair glycolysis, and 5 mM NaCN to block the mitochondrial electron transport chain [25].

### Quantitative Digital-Imaging Fluorescence Microscopy

H9c2 cells were cultured in 4-well chamber slides (Nalge Nunc International, Naperville, IL, USA). After treatment, cells were fixed with 4% paraformaldehyde for 10 min, permeabilized with 0.2% (v/v) Triton X-100 in PBS for 10 min and subsequently washed with 0.05% (v/v) Tween-20 in PBS. Next, cells were incubated for 1 h at room temperature (RT) and then overnight at 4 °C with the primary antibodies; rabbit anti-human FOXO1 (1:100, Cell Signaling Technology, Danvers, USA), goat anti-human p47^phox^ (1:50, Santa Cruz Biotechnology, Santa Cruz, CA, USA), rabbit anti-nitrotyrosine (1:50, Invitrogen, Eugene, OR, USA), an indirect marker for ROS production. After a wash with 0.05% (v/v) Tween-20 in PBS, cells were incubated for 30 min at RT in the dark with the secondary antibodies; donkey-anti-rabbit- Cy5 (1:40, Alexa Fluor 647, Invitrogen) or donkey-anti-goat-Cy3 (1:40, Alexa Fluor 568, Invitrogen). All antibodies were diluted in Normal Antibody Diluent (Immunologic, Duiven, The Netherlands). After a wash in 0.05% (v/v) Tween-20 in PBS, the slides were covered using 4′,6-Diamidino-2-Phenylindole (DAPI)-containing mounting medium (Vector Laboratories, Burlingame, CA, USA). Controls using only the secondary antibodies all showed no staining (data not shown).

The slides were analyzed with a 3I Marianas^TM^ digital-imaging microscopy workstation (Zeiss Axiovert 200 M inverted microscope; Carl Zeiss, Sliedrecht, The Netherlands) equipped with a nanostepper motor (*Z*-axis 10 nm) multiple brightfield and darkfield imaging modalities and a thermo-electrically cooled EMCCD camera (QuantEM: 512 C, 512 × 512 pixels; Photometrics, Tucson, AZ, USA). Data acquisition as well as data processing were performed using Slidebook^TM^ software (version 4.2; Intelligent Imaging Innovations, Denver, CO, USA).

### ShRNA Transfection

The rat p47^phox^-specific RNAi sequence 5′-CCCATCATCCTTCAGACCTAT-3′ targeting nucleotides 477–497 of the rat *Ncf1* gene encoding p47^phox^ (defined by NCBI RefSeq NM_053734.2) or the non-targeting sequence 5′-CAACAAGATGAAGAGCACCAA-3′ (as a negative control) were cloned into the pLKO.1 shRNA expression vector [26]. pLKO.1 plasmids express 52 basepair shRNA molecules with 21-nucleotide mRNA specificity, driven by the efficient, ubiquitously active U6 snRNA promoter. Two additional p47^phox^-specific RNAi sequences targeting nucleotides 669–719 or 86–106 were also tested, but showed lower knockdown efficiency (results not shown). Cells were transiently transfected with Lipofectamine (Invitrogen), according to the manufacturers’ protocol. Briefly, 0.8 μg DNA was mixed with 2.0 μl Lipofectamine in 500 μl DMEM without serum and incubated for 20 min at RT, after which the transfection mix was applied to 90% confluent cells. A total of 72 h after transfection, media were removed and cells were incubated with MI buffer for 4 h.

### Western Blotting

After treatment, the nucleus and cytosol fractions of H9c2 cells were separated using the NE-PER Nuclear and Cytoplasmic Extraction Kit (Thermo Scientific, Rockford, IL, USA) according to the manufacturers’ protocol. Equal amounts (20 μg) of protein were dissolved in Laemmli sodium dodecyl sulfate (SDS) sample buffer, stirred and heated at 95 °C for 10 min. Proteins were separated using 10% (w/v) SDS polyacrylamide gel electrophoresis, transferred onto nitrocellulose membranes and immunoblotted using 1 h incubation at RT and then overnight at 4 °C with rabbit anti-human FOXO1 (1:1000, Cell Signaling Technology) or goat anti-human p47^phox^ (1:500, Abnova, Heidelberg, Germany). After a wash, the blots were incubated for 30 min at RT with mouse anti-rabbit-HRP or swine anti-goat-HRP (1:500, Dako, Glostrup, Denmark), subsequently washed again and visualized by enhanced chemiluminescence (ECL; 1:40, Amersham Biosciences, Uppsala, Sweden). Protein bands were quantified using a charge-coupled device camera (Fuji Science Imaging Systems, Düsseldorf, Germany) in combination with AIDA Image Analyzer software (Isotopenmessgeräte, Staubenhardt, Germany). To confirm successful cellular separation, the nuclear lamina marker Lamin B1 was used. This showed no band in the cytosolic fraction whereas a clear band was observed in the nuclear fraction (data not shown).

### Statistics

The statistical analysis was performed using Statistical Packages for Social Sciences software (IBM SPSS 22.0.0.0 for Windows, IBM, Armonk, NY, USA). Values were tested for normality and data are presented as relative percentage showing mean ± SD. To evaluate whether observed differences were significant, *t*-tests or one-way ANOVA with post hoc Bonferroni tests were used. A two sided *P*-value <0.05 was considered to be significant.

## Results

### MI Increases Nuclear p47^phox^ and ROS Production

**Fig. 1:**
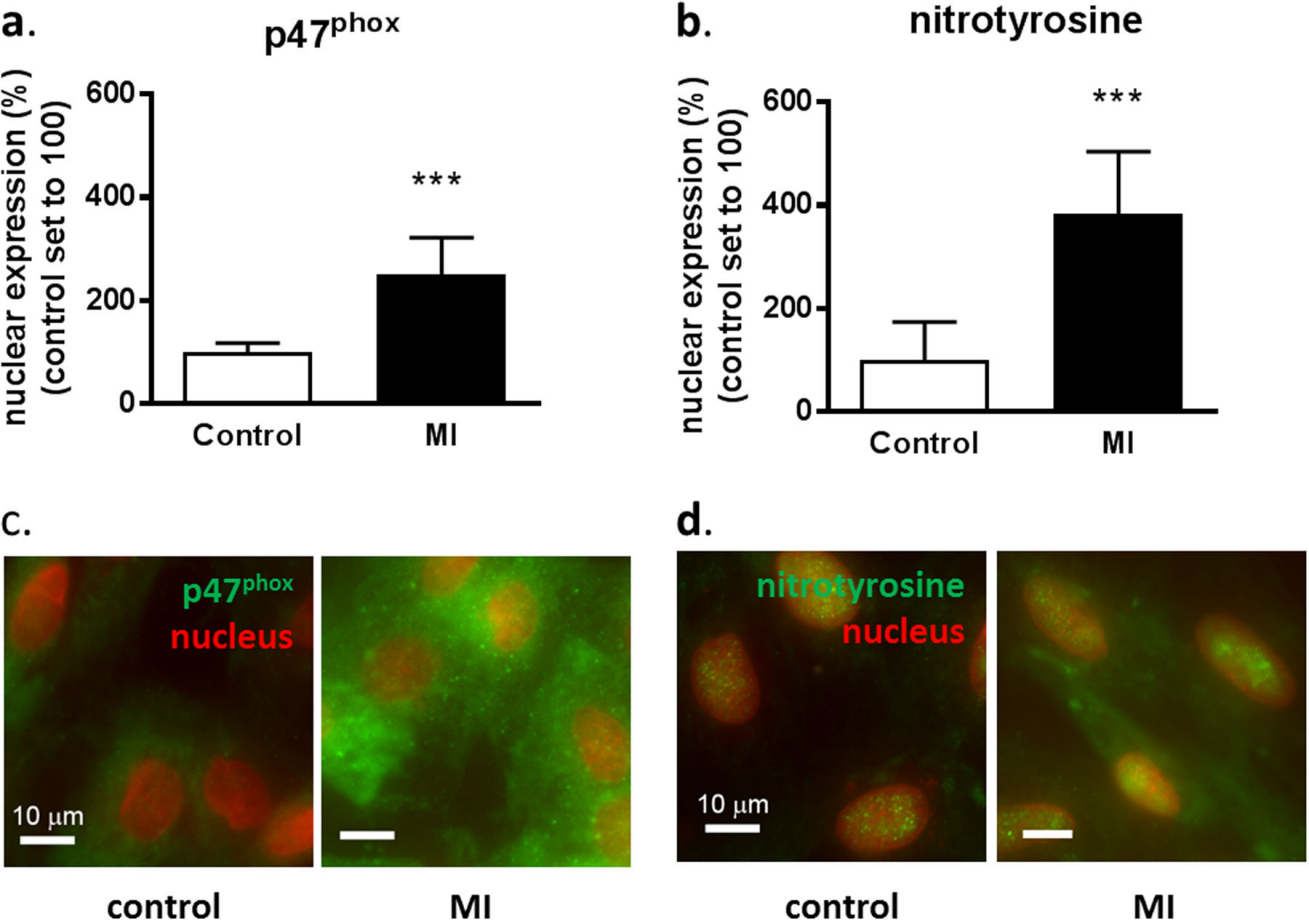
MI increases nuclear p47^phox^ expression of and nuclear nitrotyrosine production. Quantitative fluorescent digital-imaging analysis of p47^phox^ **a** and nitrotyrosine production **b** in the nucleus of H9c2 cells under control conditions or after 4 h of MI. Representative images under control and MI condition are shown in **c** of p47^phox^ and **d** nitrotyrosine. The expressions are depicted as the percentage relative to non-MI control conditions (*n* = 4). The average expression level under control conditions was set to 100%. (***P < 0.001). MI metabolic inhibition. Scale bars represent 10 µm

MI-induced nuclear translocation of p47^phox^ and local ROS production was determined using fluorescent digital-imaging microscopy of H9c2 rat cardiomyoblasts [22]. Following 4 h of MI, nuclear p47^phox^ expression was significantly higher (252 ± 70%) as compared to non-MI conditions (100 ± 17%) (Fig. 1a, c; *P* < 0.001). In addition, nitrotyrosine production was increased to 382 ± 121% following MI as compared to non-MI conditions (100 ± 74%) (Fig. 1b, d; *P* < 0.001).

### Cytosolic FOXO1 Translocates to the Nucleus Following MI

**Fig. 2:**
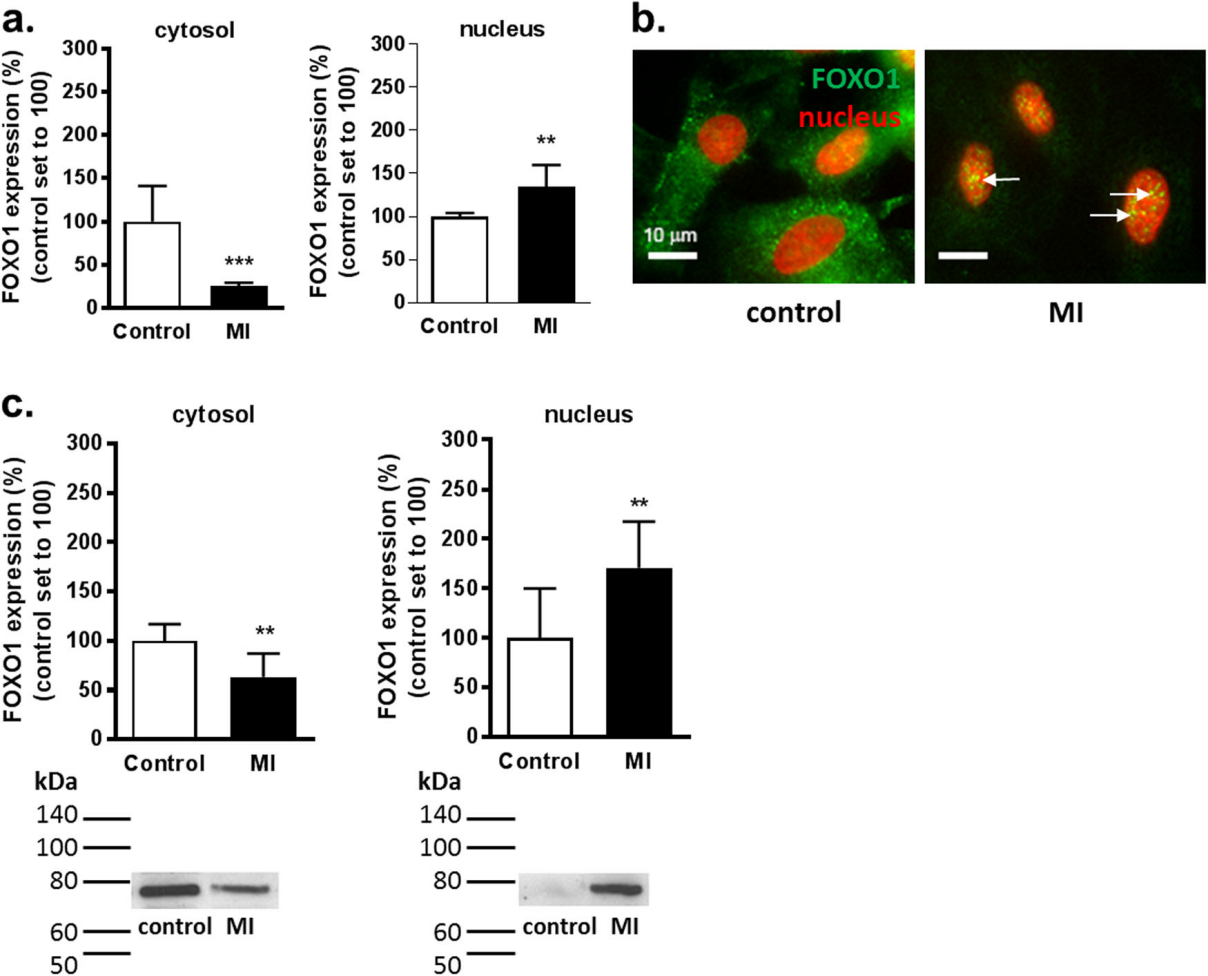
MI induces FOXO1 nuclear translocation. Analysis of the subcellular localization of FOXO1 in H9c2 cells subjected to control conditions or 4 h MI, using quantitative fluorescent digital-imaging microscopy and western blot. **a** Quantification of fluorescent digital-imaging analysis of FOXO1 expression in the cytosol and the nucleus depicted as the percentage expression levels relative to non-ischemic control conditions (*n* = 7). The average expression level under control conditions was set to 100%. **b** Example of the subcellular localization of FOXO1 (green signal). Nuclei were stained with DAPI (red signal). Arrows indicate dispersed expression of FOXO1 in the nucleus (right picture). Scale bar represents 10 µm. **c** Quantification of western blot analysis of FOXO1 in the cytosolic and nuclear fractions are presented as the expression levels in percentage relative to non-MI control conditions (*n* = 4). The average expression level under control conditions was set to 100%. Statistical (**P < 0.01; ***P < 0.001). MI metabolic inhibition

To study the effect of ischemia on the subcellular localization of FOXO1 expression in H9c2 cells, quantitative digital-imaging fluorescence microscopy was used. Under control conditions, expression of FOXO1 was mainly located in the cytosol of H9c2 cardiomyoblasts with occasional nuclear expression (Fig. 2a, b). When subjected to MI, cytosolic FOXO1 expression decreased to 25 ± 4% in comparison to non-MI condition (*P* < 0.001, Fig. 2a) whereas nuclear FOXO1 expression increased to 135 ± 25% (*P* < 0.01, Fig. 2a). These results were validated using western blot analysis of isolated nuclear and cytosol fractions of H9c2 cells. This showed that following MI, cytosolic FOXO1 protein expression level was reduced to 63 ± 24% (Fig. 2c; *P* < 0.01). Nuclear FOXO1 expression was initially low under control conditions and this was increased after 4 h of MI 171 ± 47% (Fig. 2c; *P* < 0.01). Immunoblotting of Lamin B1, an intermediate-filament protein of the nuclear lamina, was performed to ensure successful separation of the nuclear fraction. This revealed a thick band in the nuclear fractions whereas cytosolic fractions showed no band (data not shown).

### Inhibition of p47^phox^-Mediated ROS Production Reduces Nuclear FOXO1

To study the effect of p47^phox^-mediated ROS production on the FOXO1 subcellular localization, p47^phox^ was knocked-down using shRNA and cells were subjected to MI. First, the effectiveness of the p47^phox^-specific shRNA on the expression of p47^phox^ protein was validated by western blot and quantitative digital-imaging fluorescence microscopy.

**Fig. 3:**
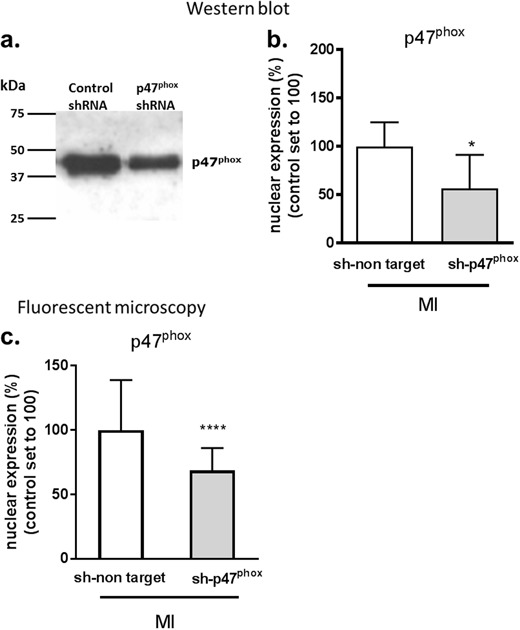
p47^phox^ knockdown in MI challenged H9c2 cells. Efficacy validation of p47^phox^-specific shRNA transfection of H9c2 cells on the expression of p47^phox^. **a** Western blot analysis of cellular p47^phox^ protein levels in cells transfected with either a p47^phox^-specific shRNA (sh-p47^phox^) or a shRNA carrying a non-targeted RNA sequence (sh-non target) and subsequently subjected to 4 h of MI (*n* = 3) and quantitatively presented in **b**. **c** Quantification of fluorescent digital-imaging microscopy of nuclear p47^phox^ expression in cells transfected with either sh-p47^phox^ or sh-non target and subsequently subjected to 4 h of MI. Expressions are depicted as the expression levels in percentage relative to non-MI control conditions (*n* = 3). The average expression level under control conditions was set to 100%. (*P < 0.05; ****P < 0.0001). MI metabolic inhibition

Western blot analysis showed that transfection of H9c2 cells with p47^phox^-shRNA significantly decreased the p47^phox^ protein expression to 56 ± 35% as compared to cells transfected with a control shRNA both following MI (100 ± 25%) (Fig. 3a, b; *P* < 0.05). Equal amounts of total protein were loaded into the gel preceding the western blot. Additionally, fluorescent microscopy then also showed a significant decrease in nuclear p47^phox^ expression to 69 ± 17% in cells transfected with p47^phox^-shRNA as compared to cells transfected with control shRNA (100 ± 39%) following MI (Fig. 3c; *P* < 0.0001). These results show that p47^phox^ protein expression was successfully knocked-down using p47^phox^-shRNA.

**Fig. 4:**
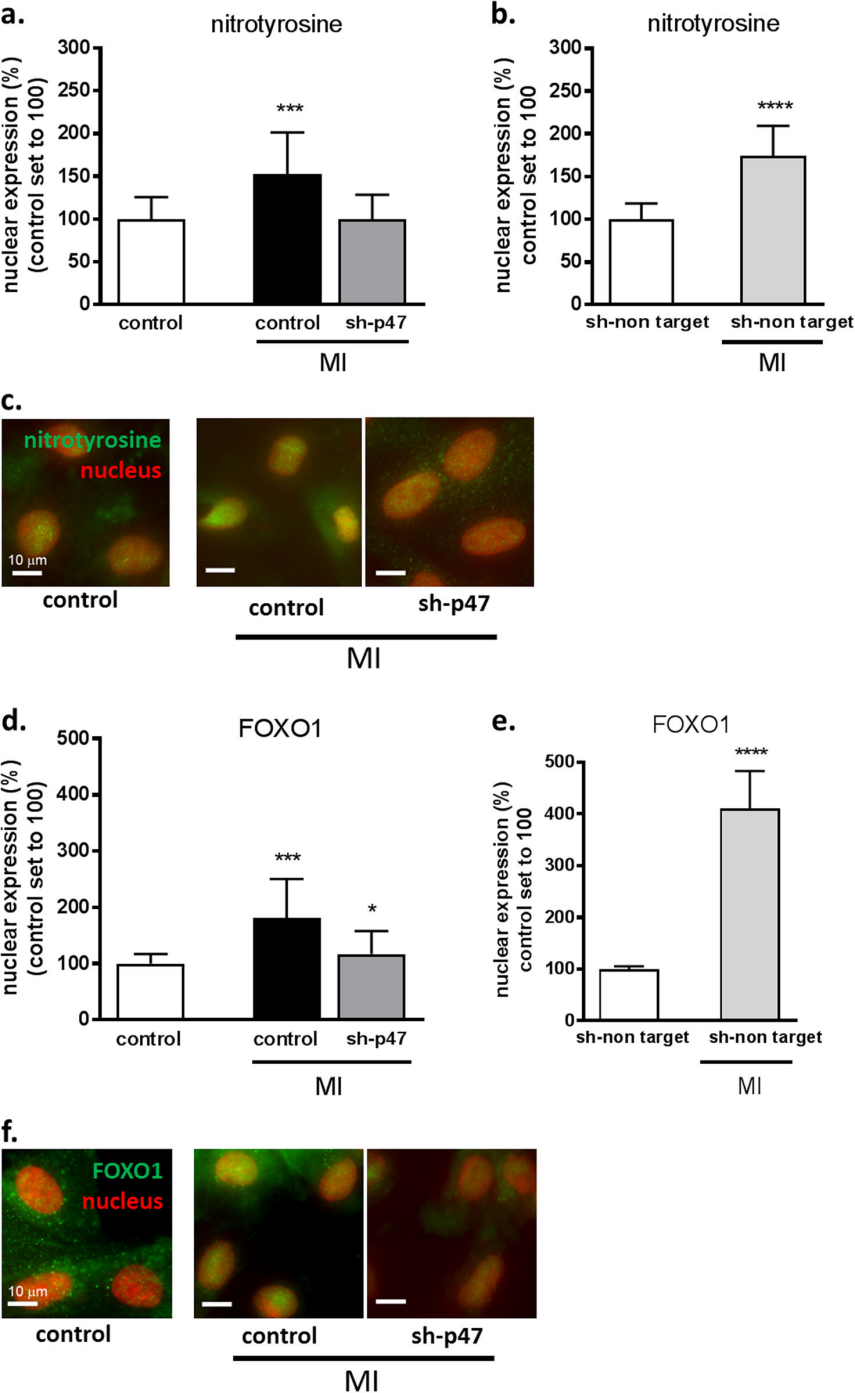
p47^phox^ knockdown prevents MI-induced nuclear nitrotyrosine production and FOXO1 expression. Quantitative fluorescent digital-imaging analysis of nuclear nitrotyrosine production (**a**) or FOXO1 (**d**) expression in H9c2 cells under control conditions or by 4 h of MI either or not preceded by p47^phox^-specific shRNA transfection (sh-p47^phox^). Representative images of nitrotyrosine production (**c**) and FOXO1 (**f**) under control conditions and following MI, and the effect of sh-p47^phox^ hereon. Expression levels of nuclear ROS production (**b**) or FOXO1 (**e**) expression in H9c2 cells transfected with a shRNA carrying a non-targeting RNA sequence (sh-non target) subjected to either non-MI control conditions or 4 h of MI. Expressions are depicted as the expression levels in percentage relative to non-MI control conditions. The average expression level under control conditions was set to 100%. (*P < 0.05; ***P < 0.001; ****P < 0.0001). MI metabolic inhibition. Scale bars represent 10 µm

Subsequently, we showed that following MI, knockdown of p47^phox^ abolished the MI-induced increase of nuclear nitrotyrosine production and showed nuclear nitrotyrosine levels (100 ± 29%) comparable to non-MI conditions (100 ± 26%) (Fig. 4a, c). Furthermore, in cells transfected with a non-targeting shRNA, nuclear nitrotyrosine production was still significantly increased following MI (153 ± 49%) as compared to non-MI conditions (100 ± 19%; *P* < 0.0001), indicating that the transfection procedure alone did not affect nitrotyrosine production (Fig. 4b).

Additionally, the MI-induced increase in nuclear FOXO1 expression (182 ± 69%) was also prevented by knockdown of p47^phox^, resulting in comparable nuclear FOXO1 expression (116 ± 42%) as under non-MI conditions (100 ± 17%) (Fig. 4d, f). Moreover, cells transfected with a non-targeting shRNA sequence responded similar to MI as cells without transfection and showed an significantly increased nuclear FOXO1 expression (410 ± 73%) in comparison to non-MI conditions (100 ± 5%; *P* < 0.0001) (Fig. 4e).

## Discussion

Though it has been well established that ROS can be involved in the activation of FOXO transcription factors, the cellular source (s) of these ROS remains largely unknown. In this study, we used nitrotyrosine as an indirect marker for ROS production, which has been shown to correlate with H_2_O_2_ production [23, 27–29]. We show for the first time that ROS produced by p47^phox^ are critically involved in the nuclear translocation of FOXO1 following MI in H9c2 cells. The subcellular localization of FOXO is essential for its activity in response to external stimuli [15, 30]. Once activated following ischemia, FOXO has been described to upregulate genes involved in pathways concerning cellular longevity or apoptosis [15, 31, 32]. Thus, with the current study we contribute to expanding knowledge about proteins involved in the regulation of FOXO activation and thereby increases insight on possible mechanism that are involved in regulating cellular fate following ischemia. We have shown earlier in ischemia challenged H9c2 cells, that ROS production at the nuclear pore is p47^phox^-dependent. This nuclear localization is hypothetically an ideal site to influence protein nuclear translocation [22].

The exact mechanism by which the p47^phox^-induced ROS production regulates FOXO1 subcellular translocation may involve multiple mechanisms and is therefore complex. Insulin and growth factors have widely been shown to affect the phosphorylated state of kinases such as ERK or Akt which in turn regulate FOXO phosphorylation and thereby nuclear trafficking and activation [13, 31]. On the other hand, oxidative stress factors have also been shown to regulate FOXO1 localization, either directly through FOXO modification or indirectly by affecting ERK and Akt [16]. Whether p47^phox^-induced ROS production regulates the nuclear translocation of FOXO through direct or indirect mechanisms remains to be established. It is most likely that the NOX2 isoform is involved in our observed p47^phox^-induced nuclear translocation of FOXO1 and increased nuclear ROS production. This because p47^phox^ is predominantly involved in NOX2 activation [33]. Moreover, p47^phox^ was shown to co-localize with NOX2 and ROS in ischemic H9c2 cells, which strongly implicates the involvement of p47^phox^ in NOX2-induced ROS production. [22, 34]. However, it has also been suggested that the p47^phox^ is not specific for NOX2 [35]. In mouse endothelial cells it has been demonstrated in vivo that p47^phox^ is also involved in NOX1 activation [35]. Although a specific relation of NOX1 and p47^phox^ activation following ischemia in cardiac cells in particular has not been reported.

In absence of p47^phox^ expression through knockdown, we showed that the MI-induced FOXO1 translocation and ROS production was abolished. This implicates an important link between activation of NOX2 proteins and their production of ROS which thereby alters FOXO1 subcellular location. Although NOX proteins have been shown to mediate redox-sensitive signal transduction, only a few other studies have demonstrated a direct link between NOX proteins and their activation of FOXO transcription factors as shown in the current results [20, 21]. In cultured human pulmonary artery SMC, an essential role for NOX4 in the activation of FOXO3a was shown [20]. Another study showed a reduction in the inactivation of FOXO4 transcriptional activity in hepatic stellate cells (HSC) cultured from NOX1-knockout mice [21]. The results of these two studies indicate that NOX proteins affect FOXO activity, but also that NOX isoforms may have opposing effects. Whether the various NOX isoforms control different FOXO family members or to what extent species, cell type, or (patho)physiological stimulus determines their specific involvement and activity remains to be determined.

The exact function of FOXO1 function in response to ischemia in cardiomyocytes or other cell types is under debate. Post translational modifications of FOXO1 following ischemia that result in nuclear translocation and activation following ischemia have been well described in the mouse and rat cardiomyocytes [17, 36, 37] and in H9c2 cells [38], but also in the mouse liver [39], [17, 36, 37] and in gerbil and mouse neurons [38, 40]. However, once activated, FOXO1 can either exert a pro-apoptotic [37, 39–41] or an anti-apoptotic effect [14, 17, 36, 38]. In neuronal cells, nuclear FOXO1 can activate the Fas ligand and BIM gene promoting apoptosis [40]. This suggests that cytoplasmic phosphorylation and thereby inactivation of FOXO1 would contribute to cell survival. Additionally, in mouse follicular granulosa cells it has been shown that FOXO1 nuclear translocation was enhanced following oxidative stress through increased c-Jun N-terminal protein kinase activity and thereby contributed to apoptosis [41]. In contrast, Sengupta et al. [17] showed in FOXO1/FOXO3 double null-mutant mice that increased myocardial oxidative damage together with a diminished myocardial function was observed following acute ischemia-reperfusion injury, in comparison to wild-type mice [17]. As the authors suggest, this most likely pointed to a FOXO-mediated cardioprotective mechanism through initiating the expression of antioxidant, anti-apoptotic and autophagy genes in response to oxidative stress [17]. This hypothesis has been supported by other studies showing that following oxidative stress, nuclear FOXO contributes to an upregulation of antioxidant genes such as superoxide dismutase and catalase [16, 36, 38, 42]. The exact mechanism by which FOXO1 functions following ischemia in cardiomyocytes in vivo and in vitro and the exact role of p47^phox^ herein remains to be determined. However, FOXO1 appears to respond to multiple cell stress and survival signals and, depending on the presence and magnitude of these signals, p47^phox^-induced ROS production and FOXO1 may be critically involved in cell fate decisions following ischemia.

## Conclusion

Following ischemia, ROS are important mediators in cell fate decisions and have been demonstrated to influence the activity of FOXO transcription factors. With the current study for the first time we gain insight in the cellular sources of these ROS. MI challenged H9c2 cells showed an increase in nuclear nitrotyrosine production and FOXO1 translocation which were both abolished when we knocked-down p47^phox^. In an earlier study, we have shown that nuclear ROS production is p47^phox^-dependent and co-localizes with NOX2 in ischemia challenged H9c2 cells. Together with the results in the current study, this indicates that in ischemia or MI challenged H9c2 cells, FOXO1 nuclear translocation critically depends on p47^phox^/NOX2-induced ROS production.
